# Elevated Contaminants Contrasted with Potential Benefits of ω-3 Fatty Acids in Wild Food Consumers of Two Remote First Nations Communities in Northern Ontario, Canada

**DOI:** 10.1371/journal.pone.0090351

**Published:** 2014-03-05

**Authors:** Timothy A. Seabert, Shinjini Pal, Bernard M. Pinet, Francois Haman, Michael A. Robidoux, Pascal Imbeault, Eva M. Krümmel, Linda E. Kimpe, Jules M. Blais

**Affiliations:** 1 Department of Biology, University of Ottawa, Ontario, Canada; 2 Indigenous Health Research Group, University of Ottawa, Ottawa, Ontario, Canada; 3 Behavioural and Metabolic Research Unit, Faculty of Health Sciences, University of Ottawa, Ottawa, Ontario, Canada; INRA, France

## Abstract

Indigenous communities in Boreal environments rely on locally-harvested wild foods for sustenance. These foods provide many nutritional benefits including higher levels of polyunsaturated fatty acids (PUFAs; such as ω-3) than what is commonly found in store-bought foods. However, wild foods can be a route of exposure to dietary mercury and persistent organic pollutants (POPs) such as polychlorinated biphenyls (PCBs). Here, we show a strong association between the frequency of wild food consumption in adults (N = 72) from two remote First Nations communities of Northern Ontario and environmental contaminants in blood (POPs) and hair (mercury). We observed that POPs and mercury were on average 3.5 times higher among those consuming wild foods more often, with many frequent wild food consumers exceeding Canadian and international health guidelines for PCB and mercury exposures. Contaminants in locally-harvested fish and game from these communities were sufficiently high that many participants exceeded the monthly consumption limits for methylmercury and PCBs. Those consuming more wild foods also had higher proportions of potentially beneficial ω-3 fatty acids including eicosapentaenoic acid (EPA) and docosahexaenoic acid (DHA). These results show that the benefits of traditional dietary choices in Boreal regions of Canada must be weighed against the inherent risks of contaminant exposure from these foods.

## Introduction

Elevated contaminant levels in wild foods have been well documented in Canadian indigenous communities, particularly in northern Inuit populations, where higher contaminant exposures can be related to the consumption of certain marine mammals [1]. These contaminants include mercury and persistent organic pollutants (POPs) such as organochlorine pesticides, polychlorinated biphenyls (PCBs), and more recently polybrominated diphenyl ethers (PBDEs) [2]. In remote northern Boreal regions of Canada, environmental contaminants are generally considered to be at low concentrations, with the exception of mercury [3]. Elevated concentrations of mercury can be found in aquatic biota, particularly those in acidic, dystrophic lakes [1]. When mercury (Hg) is microbially transformed to methylmercury (MeHg), it can cross the blood-brain and placental barriers in humans, harming the brain and nervous system even at low exposure levels, particularly in the developing nervous system of a fetus or young child [4]. Recently, MeHg exposure has also been linked to symptoms of attention deficit/hyperactivity disorder (ADHD) among Inuit children [5].

Despite the potential presence of contaminants in local wild food, their consumption is often promoted as a preventive measure for deleterious health problems [2], [6], [7]. Fish, in particular, continues to be promoted as a healthy food choice for First Nations people [8]. Wild foods are significant sources of dietary protein, essential minerals [9], and very long-chain (VLC) omega-3 polyunsaturated fatty acids [10]–[12]. The VLC ω-3 fatty acids, such as eicosapentaenoic acid (EPA) and docosahexaenoic acid (DHA), have potential protective benefits against cardiovascular disease, obesity and diabetes [13], [14] which continue to be prevalent in indigenous populations [15]–[17]. Fish oil supplements may also provide these benefits, though the source of these fish oils must be carefully considered [18] due to their potential contamination by POPs.

Northern First Nations peoples are gradually shifting away from local wild food sources [19], [20] and concurrently increasing their consumption of less nutritious store-bought foods. These foods are high in caloric content, carbohydrates, and saturated fats and lack important micronutrients and VLC ω-3 fatty acids [13]. The average daily intake of polyunsaturated fatty acids (PUFAs) in the typical North American diet is 34 g/day with VLC ω-3 fatty acids making up only 0.5% of total PUFAs [21]. Conversely, eating exclusively off the land has the potential to provide a total PUFA intake of 27 g/day, 6% of which would be VLC ω-3 fatty acids, as estimated by hunter-gatherer societies' studies [22].

Therefore, while the greater nutrient and fatty acid diversity of wild foods is promoted in dietary recommendations, the possible exposure to contaminants contrasted with the potential nutritional benefits of wild food consumption presents a dilemma which needs to be considered in the context of region-specific food consumption patterns (both quantity and variety). Our objectives here were to: 1) compare contaminant exposure in two remote First Nation communities through dividing participants into categories based on frequency of wild food consumption; and 2) relate contaminant exposure to plasma ω-3 PUFA levels. The contrast of contaminant exposure and potential nutritional benefits has implications for risk assessments and food consumption advisories as well as the prevention and treatment of chronic diseases.

## Materials and Methods

### 2.1 Participant recruitment and dietary surveys

**Figure 1:**
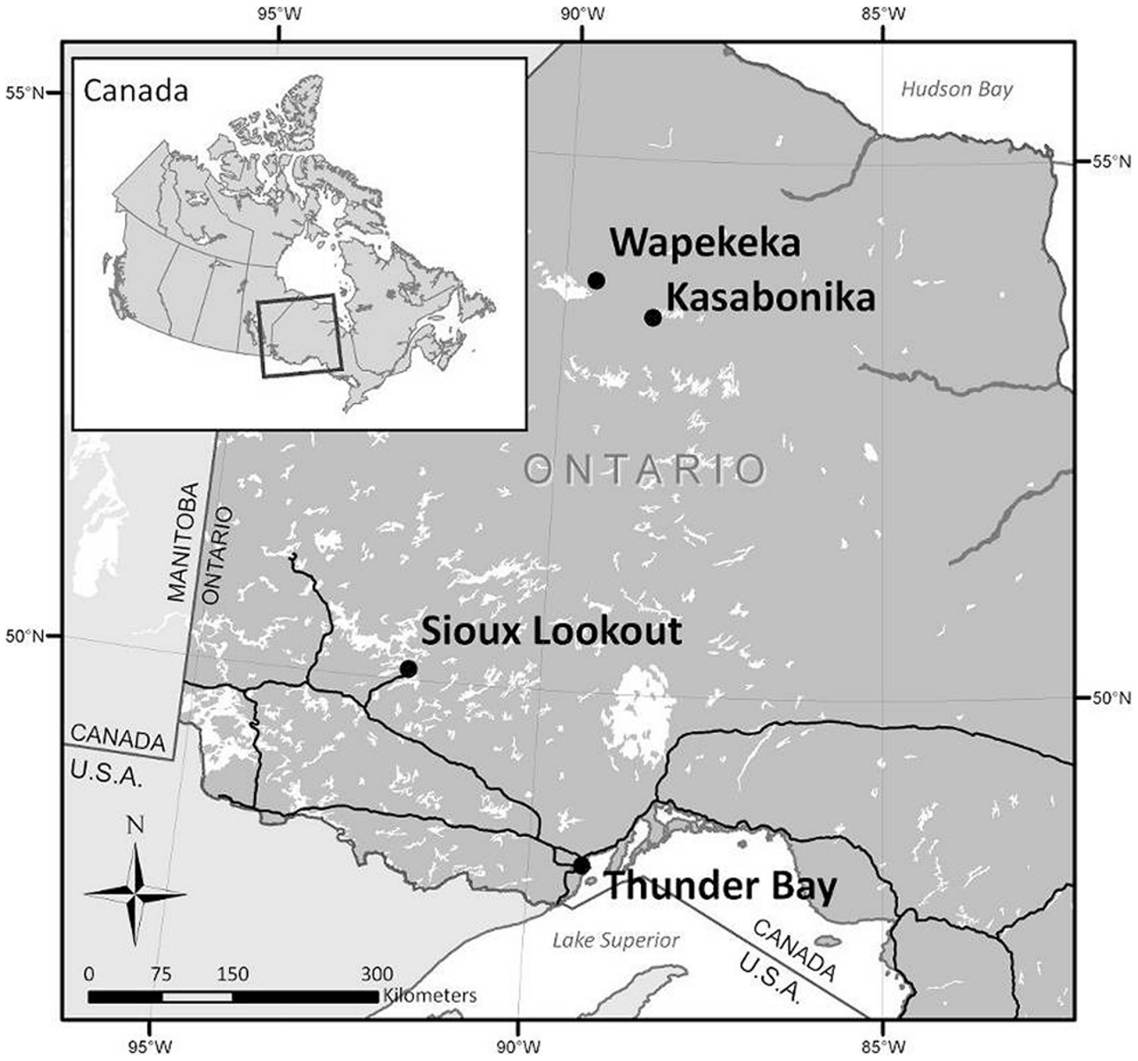
Map showing Wapekeka and Kasabonika First Nations in northern Ontario, Canada (inset).

All participants provided written consent to participate in this study using protocols approved by the University of Ottawa Research Ethics Board and Health Canada Ethics Board. Participant recruitment was conducted in Wapekeka and Kasabonika Lake First Nations (Figure 1) in September 2007. A purposeful sampling research design [23] was used to recruit participants based on dietary extremes, with participants either eating predominantly land-based foods (primarily wild game) or eating predominantly market foods. Following recruitment procedures as defined by local band councils and health officials, local research coordinators were asked to identify people in each community who were thought to eat primarily wild food or market food. Potential participants were then contacted by local coordinators to determine if they would be willing to be interviewed about their dietary habits. Informed consent was obtained from each participant following guidelines approved by the University of Ottawa Research Ethics Board and Health Canada Ethics Board. The interviews, which were semi-structured, lasting between 20 and 45 min, focused on wild food consumption frequency, what types of species are eaten and parts of the animals are eaten. Food frequency was considered in light of seasonal variation and harvesting patterns which had clear effects on wild food consumption.

The number of individuals that participated from each community (39 in Wapekeka and 33 in Kasabonika) represented approximately 9% of the eligible population in Kasabonika and 24% of the eligible population in Wapekeka at the time of the study. Although samples were collected from 72 participants, analyses based on diet were conducted on the data of 71 subjects due to incomplete dietary information of one participant. Based on individual responses and willingness to participate in the study, participants were assigned an identification code to ensure anonymity and confidentiality and were categorized based on their levels of wild food consumption (WF1, WF2, and WF3). The WF1 group (n = 28) consisted of individuals who ate one wild food meal per month or less. The WF2 group (n = 22) included those who ate less than one wild food meal per week but more than one per month. The WF3 group (n = 21) consisted of those who ate more than one wild food meal per week. Additional inclusion criteria required that individuals be First Nation, over 18 years of age, non-pregnant, and free of type 1 diabetes. The food categories created based on the semi-structured interviews have been verified using isotopic enrichment in ^13^C and ^15^N as well as differences in fatty acid profiles in circulating phospholipids [24]. Results from that study showed that participants consuming wild foods at least once a week showed lower ^13^C enrichment (in plasma and in hair) but higher (nitrogen) isotope ratios. In addition, frequent wild food consumers showed higher contributions of arachidonic acid (20∶4 ω-6 and docosahexaenoic acid (DHA, 22∶6 ω-3) to total phospholipid fatty acids and lower linoleic acid (18∶2 ω-6).

### 2.2 Plasma Sample collection from participants

Blood (n = 72) and hair (n = 71) samples were collected from participants during the peak harvesting/wild consumption period in October and November 2007. Based on extensive ethnographic field work [20], the fall season was purposively chosen as it was determined to be the peak hunting season and many wild foods are collected and consumed at this time of year. Mercury levels are known to fluctuate throughout the year depending on exposure. For example a study on fishermen showed that their hair mercury levels doubled during the fishing season [25]. Thus, to capture the most likely time of highest contaminant exposure, hair in our study was sampled during the hunting season. As organic pollutants are known to bioaccumulate in adipose tissue, POP concentrations are mobilized during fasting and blood levels can fluctuate accordingly (e.g. [26]). However, blood contaminant levels can also be expected to fluctuate based on a meal being digested. To allow for a more integrated contaminant signal (i.e. one that was not based on immediate dietary intake) blood samples were obtained after individuals had fasted for 8 hours. Blood was collected from an antecubital vein and put into vacutainer glass tubes (Becton, Dickinson and Company) containing ethylenediaminetetraacetic acid (EDTA). Tubes were immediately centrifuged at 3500 revolutions per minute (1917 g) for 10 minutes. Plasma was transferred into sterile microcentrifuge tubes (Fisher Scientific) and kept at −20°C for safe shipping to the University of Ottawa. Upon receipt, plasma samples were stored at −80°C for future analyses. Hair samples were collected from the nape of the neck as close to the scalp as possible using stainless steel scissors. Samples were sealed separately in labelled bags for safe transportation to the University of Ottawa. Hair was not collected from one of the participants in Wapekeka, hence the discrepancy between the number of blood (n = 72) and hair (n = 71) samples.

Anthropometric measurements included body weight, height, and waist circumference and were taken prior to blood and hair collection. Body weight was measured using a standard beam scale, and height and waist circumference were determined with a measuring tape. Height was measured with the feet together, having the heels, back, and head against a wall, and following normal inspiration. Waist circumference was measured directly on the skin, in duplicate and averaged, at the mid-point between the last floating rib and the top of the iliac crest. Body mass index (BMI) was calculated by dividing body weight (kg) by square height (m^2^).

### 2.3 Analysis of plasma samples for POPs

The organic pollutants chosen for analysis in this study were those most likely to exceed guidelines set by the World Health Organization (WHO) and Health Canada. Contaminants determined in blood (plasma) were Aroclor 1260, PCB congeners PCB28, PCB52, PCB99, PCB101, PCB105, PCB118, PCB128, PCB138, PCB153, PCB156, PCB163, PCB170, PCB180, PCB183, PCB187, aldrin, α-chlordane, γ-chlordane, β-hexachlorocyclohexane (β-HCH), *cis*-nonachlor, *trans*-nonachlor, *p,p*'-dichlorodiphenyldichloroethylene (*p,p*'-DDE), dichlorodiphenyltrichloroethane (DDT), hexachlorobenzene (HCB), mirex, oxychlordane, polybrominated biphenyl (PBB)153, PBDE congeners PBDE47, PBDE99, PBDE100, PBDE153, toxaphene parlar26, and toxaphene parlar50. Aroclor 1260 was calculated as follows:

Aroclor 1260 concentration = (C153+C138) * 5.2 where: C153 = PCB 153 concentration and C138 = PCB 138 concentration [27].

Briefly, the plasma samples were enriched with internal standards and denatured with formic acid. The compounds were extracted from the aqueous matrix using solid phase separation and extracts were cleaned by florisil column prior to analysis. The POPs were eluted from columns using methylene chloride-hexane (9 mL; 25∶75 vol/vol). The solvent was evaporated, taken up in 20 uL of hexane and analyzed for POPs on GC-MS (gas chromatography-mass spectrometry; E-446), Chromatograph 6890 (Agilent) using a solid phase extraction followed by gas chromatography coupled to mass detection (Agilent 5973 network). Peaks were identified by relative retention times obtained on the two columns using a computer program developed by the Centre de Toxicologie du Québec (Quebec Toxicology Centre). Generated ions were measured after negative chemical ionization. The concentration of each analyte measured was determined using percent recovery of isotopically labelled internal standards. To verify results, an interlaboratory comparison was made with the External Quality Assessment Scheme (G-EQUAS), Germany. Data were screened to exclude POPs that were below the detection limit in more than 60% of cases. Results of individual PCB congeners are not reported here, but rather the sum of 12 PCB congener concentrations (PCB99, PCB105, PCB118, PCB128, PCB138, PCB153, PCB156, PCB163, PCB170, PCB180, PCB183, and PCB187) is presented. Aroclor 1260 was calculated for the blood of every participant and none of the Aroclor 1260 concentrations were below the detection limit (0.038 µg/L). To reduce bias, random numbers were generated between 0 and the detection limit of each POP included in analysis for cases below the detection limit [28]. A particular POP was considered in the analysis if the detection frequency was greater than 60%. The replacement of non-detects with random numbers did not change the significance of statistical tests used during analysis. Total plasma lipid concentration (g/L) was calculated using the following formula published: TL = (2.27×TC)+TG+0.623, where TC is the concentration of total cholesterol (g/L) and TG is the concentration of triglycerides (g/L) [29]. Enzymatic tests were used for the direct quantitative measurement of cholesterol and triglycerides in plasma on Roche automated clinical chemistry analyzers.

### 2.4 Analysis of hair samples for mercury

All hair samples (n = 71) were divided into 1 centimetre (cm) lengths, starting from the base, which represented approximately one month of recent hair growth. Samples were cleaned by soaking in a 2∶1 chloroform: methanol solution to remove any residues, rinsing several times with distilled water, and thoroughly drying before any analyses were performed. For total mercury analysis, 2–5 mg of hair (1 cm lengths from the scalp) was placed in a mercury SP-3D analyzer (Nippon Instruments Corporation, Japan) which heated the samples to a maximum temperature of 950°C. Mercury released from the hair was subsequently collected and isolated in a two-stage gold amalgam process before being transferred and detected via cold vapor atomic absorption spectroscopy. Blanks and a standard solution diluted from a stock solution of Fisher Scientific (CSM114-100) Certified Reference Material 1000 ppm for Trace Metals sample (Dorm-3, National Research Council) were included as quality controls.

### 2.5 Plasma phospholipid fatty acids analysis

Circulating lipids were extracted twice in chloroform-methanol (2∶1 v/v) from 200 µL of plasma collected in the fasting state according to the Folch method [30]. Extracted lipids were then suspended in chloroform in which phospholipids were separated by filtration on Superclean solid-phase extraction tubes (1 ml LC-NH_2_; Sigma, St Louis, MO, USA) as previously described [31] and then eluted with methanol. To this, 200 µL of margaric acid (17∶0; 30 µg/100 µL hexane) was added as an internal standard and phospholipids were then trans-esterified with acetyl chloride in methanol for 2 h at 90°C. After evaporation, the newly formed fatty acids methyl ester were dissolved in 60 µL isooctane and 2 µL were injected in a Hewlett-Packard gas chromatograph (HP 9890 with HP 7683B autosampler) equipped with a flame-ionization detector and a 60 m fused silica column (DB-23; J&W Scientific, Folsom, CA, USA). The carrier gas was nitrogen and detector gases were hydrogen and air. Injection port temperature was set at 220°C and the detector at 240°C. The temperature was 185°C for 35 min after injection, raised to 210°C at a rate of 5°C min^−1^, and kept at 210°C for an additional 10 min. Exact retention times of each fatty acid were determined with pure standards (Sigma-Aldrich, St Louis, MO, USA). Plasma phospholipid (PL) concentrations were measured by colorimetry using the PL-C microtiter procedures kit (Wako Diagnostics, Chemicals, Richmond, VA). Levels for individual fatty acids were expressed as a percent contribution to total PL fatty acids content.

### 2.6 Wild food sampling for POPs and Hg

Wild food samples were collected with the participation of local hunters and focused on wild food sources documented through dietary interviews, 24 hour recalls and 3-day food diaries. Wild food samples were collected at different times of the year based on availability for hunting. Geese and ducks were sampled in the early spring whereas fish were sampled throughout the year, reflecting natural consumption patterns. Beavers were mostly sampled in the fall. All samples were cut using clean utensils and wrapped in aluminium foil pre-rinsed with acetone and hexane, and placed inside Whirlpak® bags (Fisher Scientific). For fish, species, fork length, caudal length, and weight were recorded. One fish fillet (skin included) was taken per fish sampled. Organs and roe (where applicable) were collected separately for analysis. For all mammalian and avian samples, we recorded species, weight, and sex. These species were dissected with the help of hunters who removed the fur and also indicated what parts of each animal were consumed by community members. Dietary interviews revealed that community members consume the muscle tissue, liver, heart, and gizzard of geese and ducks. From beaver, people consumed muscle and liver, and of rabbit, the muscle, liver, and brain. All edible portions not taken for contaminant analysis were made available to hunters for consumption. Samples were stored in a −20°C chest freezer until shipping to the University of Ottawa in coolers, which were received frozen and stored at −20°C until analysis.

Wild food samples were homogenized using the Cabela Pro 450 meat grinder. Samples were ground semi-frozen through an 8 mm plate, mixed and then reground through a 4 mm plate. Samples for total mercury analysis were homogenized and freeze-dried to determine water content. Subsamples of fish and meat were analyzed in triplicate on a Mercury SP-3D analyzer (Nippon Instruments Corporation, Japan). A certified reference material (Dorm-3, National Research Council Canada) was analyzed after every 10 samples to ensure accuracy and reproducibility of the results. Detection limits were 0.01 ng mercury per sample weighing an average 30 mg, or 0.3 ng/g. Dorm-3 certified reference materials met reported mercury concentrations of 382±24 ng/g.

### 2.7 Organic contaminants in wild food

Wild food samples for organic contaminant analysis were collected as wet homogenate, mixed with Hydromatrix (Varian), and spiked with recovery standards (1,3-DBB, 1,3,5-TBB, 1,2,4,5-TTBB, d-HCH, Endrin Ketone, BZ30 and 205, Ultra Scientific). The mixture was extracted following procedures using an Accelerated Solvent Extractor 200 [32]. The sample extracts were cleaned to remove lipids [33] and then injected on two Envirogel columns (150 and 300 mm, Waters) connected sequentially to a preparative 1200 HPLC coupled with a photodiode array and fraction collector (Agilent Technologies). The collected sample fractions were evaporated to 1 mL in 2,2,4-trimethylpentane (Fisher Scientific) and further fractionated on 8 grams of silica Davisil 635 (Fisher Scientific) packed into a chromaflex column with hexane [34]. The samples were evaporated to 500 µL in 2,2,4-trimethylpentane for analysis on a 6890 Gas Chromatograph with a micro Electron Capture Detector (Agilent Technologies) [35]. One μL was injected in splitless mode on a DB-5MS 60 m, 250 µm, 0.25 µm column (J&W Scientific).

Chromatographic peaks were interpreted using Agilent Chemstation software (Rev. B.03.01). Compounds were identified by analyzing a linear set of standards and comparing their retention times with those of the sample compounds. From the standard mixtures, approximately 35 PCB congeners and 31 pesticide compounds were confirmed. Quantitative analysis was completed with the use of octachloronaphthalene as an internal standard in the standard mixtures and samples.

Analytical blanks (n = 12), which comprised Hydromatrix® and recovery standards subjected to the entire extraction and sample clean-up procedures, contained an average of 92 pg of PCBs. Recovery was 73.4%±21.3% SD for PCB 30 and PCB 205. All samples were blank subtracted and recovery corrected. Standard Reference Material (SRM2977, National Institute of Standards and Technology) was subjected to the extraction and sample clean-up procedures, and values fell within certified limits.

### 2.8 Statistical analysis

An analysis of covariance (ANCOVA) was used to adjust for age and test the combined effects of age and wild food consumption frequency on contaminant concentrations. The PUFAs were compared to wild food consumption through ANOVAs. All data were log normalized where necessary after testing the assumption of normality through application of the Shapiro-Wilk test. Results with a p-value of less than 0.05 were considered statistically significant. All statistical analyses were performed using JMP version 10 (SAS Institute Inc).

## Results and Discussion

### 3.1 Contaminant burden in association with degree of wild food consumption

**Figure 2:**
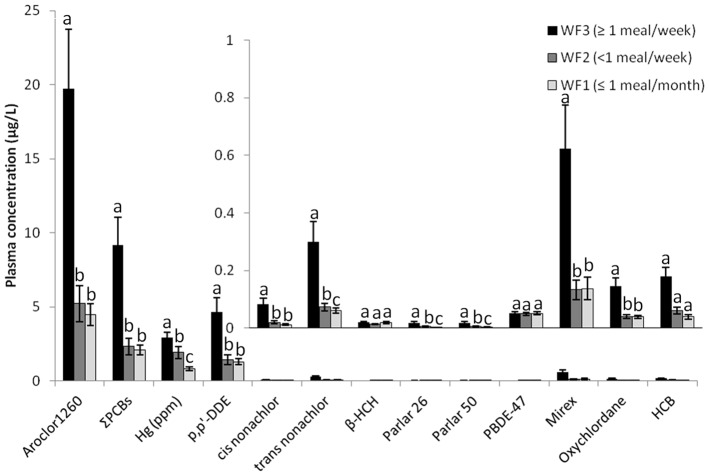
Mean (±SE) concentrations of POPs in blood (μg/L) and mercury (Hg) in hair (ppm) for participants from Wapekeka and Kasabonika First Nations consuming three levels of wild food consumption. WF1 (n = 28; ≤1 meal per month), WF2 (n = 22; <1 meal per week), and WF3 (n = 21; ≥1 meal per week). Inset is an enlargement of the concentrations for the nine contaminants on the right side of the x-axis (cis-nonachlor through HCB). Letters indicate a significant difference in groups (p<0.05) after adjustment for age. The PBDE-47 concentrations were not adjusted for age.

Participant characteristics as related to WF consumption are presented in Table 1. All contaminant concentrations except PBDE-47 were significantly correlated with age (0.0001<p<0.02), as expected [36], [37]. Once accounting for age, we observed significantly higher (p<0.05) contaminant concentrations in the WF3 group when compared toWF1 and WF2 participants for 10 of the 13 measured contaminants (Aroclor 1260, ΣPCBs, mercury, *p,p*'-DDE, *cis*-nonachlor, *trans*-nonachlor, Parlar 26, Parlar 50, Mirex, and oxychlordane; Figure 2). In addition, 4 of the 13 measured contaminants were significantly higher (p<0.05) in the WF2 group as compared to the WF1 group (Hg, *trans*-nonachlor, Parlar 26, and Parlar 50). No differences in contaminant concentrations were observed among males and females, even after adjusting for age. Health Canada's PCB guidelines indicate that the level of concern (LOC) for Aroclor1260 in human blood is 20 µg/L, and 5 µg/L for women of childbearing age [38]. Of the three WF categories, 43% of the WF3 group and 5% of the WF2 group were above the LOC of 20 µg/L. No participants in the WF1 group were above the LOC. Of the participants in our study, there were 25 women of childbearing age (18 to 45 years of age). The Aroclor1260 concentrations for these women averaged 2.5±0.38 µg/L with 4% of these women above the LOC of 5 µg/L. This result is particularly notable because epidemiological evidence has shown that children exposed prenatally to PCB concentrations can have measurable developmental and neurobehavioral deficits [5], [39]–[41]. All participants were below the WHO guideline of 200 µg/L of *p,p*'-DDT in blood.

**Table 1:** Participant characteristics (N = 71) of Kasabonika and Wapekeka First Nations based on wild food consumption frequency.

	WF1 (n = 28)	WF2 (n = 22)	WF3 (n = 21)	
**Age**	38.8 (±8.1)	40.5 (±11.3)	50.9 (±16.0)	p = 0.01
**Sex (M∶F)**	13∶15	6∶16	12∶9	–
**BMI (kg/m^2^)**	31.5 (±5.4)	30.9 (±4.2)	33.7 (±4.1)	p = 0.12
**ΣPCBs (μg/L)**	2.0 (±1.7)	2.3 (±2.5)	9.2 (±8.5)	p = 0.0002
***p,p***'**-DDE (μg/L)**	1.3 (±1.0)	1.4 (±1.6)	4.6 (±4.6)	p = 0.0001
**Mercury (ppm)**	0.8 (±0.7)	1.9 (±1.8)	2.9 (±1.6)	p<0.0001
**Ω-3 (% phospholipid FA)**	15.9 (±3.4)	20.2 (±5.2)	23.5 (±7.4)	p<0.0001
**Ω-6 (% phospholipid FA)**	37.8 (±1.8)	36.4 (±1.8)	34.8 (±3.4)	p = 0.0003

WF1 (n = 28; ≤1 meal per month), WF2 (n = 22; <1 meal per week), and WF3 (n = 21; ≥1 meal per week). Data are presented as means (±SD) where appropriate.

Exposure to POPs and Hg has been shown to trigger pro-inflammatory cytokine responses in diverse cells [42]–[46] and in cross-sectional studies as well [47]. In individuals with type 2 diabetes, chronic inflammatory marker levels have been shown to differ in concentrations [48], [49]. In this regard, we have recently highlighted that pro-inflammatory cytokine levels are elevated in First Nations people compared to Caucasians and that a small, but significant, part of this immune activation is explained by PCBs burden [50]. We have also found a significant link between POP exposure and insulin secretion and higher concentrations of certain POPs in individuals with type 2 diabetes [51].

In the past decade, several studies have shown that exposure to low levels of POPs can be detrimental. A low-dose exposure to organochlorine pesticides and PCBs has been linked to obesity [52] and type 2 diabetes [53] which are prevalent conditions among participants of this study [17]. Low-dose exposure to organochlorine pesticides has also been linked to DNA hypomethylation, which can lead to some cancers [54] and PCBs have been shown to suppress thyroid hormone receptor function, which can in turn inhibit development of the central nervous system [55].

The POP concentrations reported here were comparable to, and in some cases exceeded, those of other northern First Nations and Inuit populations [1] both for all adults (N = 72) and for women of childbearing age (n = 25; [56], [57]: Overall mean blood concentrations of *p,p*'-DDE and Mirex reported here were higher than those measured in the Inuit of Nunavik [1]. For women of childbearing age, plasma concentrations of *p,p*'-DDE and PCB153 exceeded those previously reported for Dene/Métis and Inuvik Inuit from Northwest Territories and blood concentrations of PCB153 exceeded those reported for Nunavik and Baffin Inuit [56].

Mean (±SE) age-adjusted mercury concentrations in hair were 1.5 times higher in the WF3 group (2.9±0.3 µg/g) than in the WF2 group (1.9±0.4 µg/g) and almost 4 times higher than in the WF1 group (0.8±0.3 µg/g; Figure 2). Of the participants, 95% of WF3, 67% of WF2, and 50% of WF1 had hair mercury levels that exceeded the U.S. Environmental Protection Agency's safety criterion of 1.0 µg/g mercury [58], [59]. The mean (±SE) hair mercury concentration of women of childbearing age (n = 25) was 1.3±0.3 µg/g with 44% of these women above the threshold of 1.0 µg/g. Mercury concentrations in women of childbearing age are especially important due to mercury transfer from the mother to the fetus [60]. The mean hair concentration of mercury found here was higher than those reported in two communities in the Northwest Territories: the Inuvik region and the Gwich'in community of Tsiigehtchicm [1]. The mean levels we found were however lower than the community of Tuktoyaktuk, also located in the Northwest Territories.

### 3.2 Contaminants in locally-harvested wild food samples

**Figure 3:**
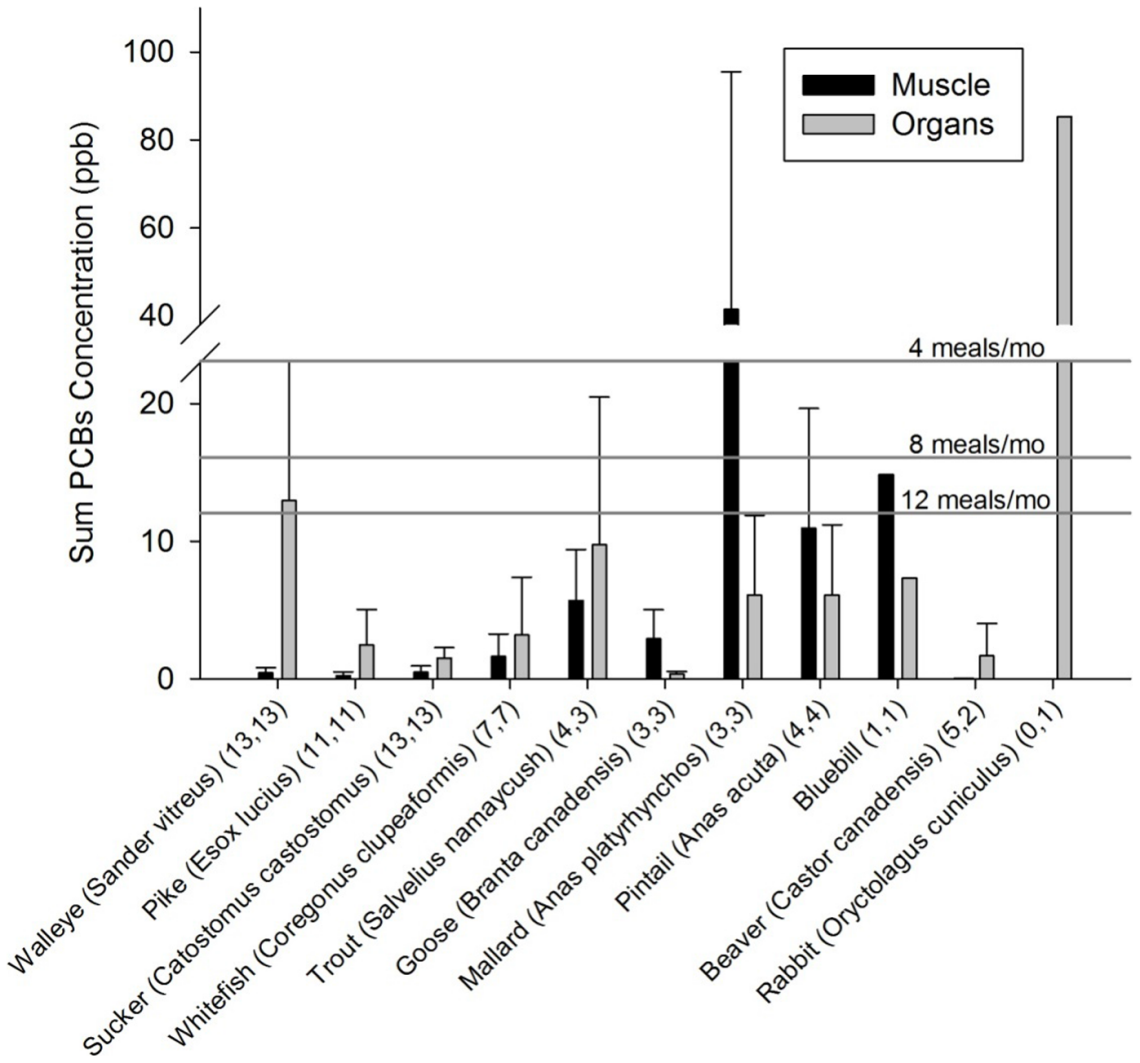
Mean (±SD) ΣPCBs concentration (ppb or ng/g fresh weight) in muscle and organs of various locally-harvested wild animals generally consumed by community members of Kasabonika and Wapekeka First Nations. Sample sizes are indicated (muscle, organ tissues) for each species. Monthly fish consumption limits for the U.S. Environmental Protection Agency are indicated. The consumption limits are 12 meals/month (12–16 ppb), 8 meals/month (16–23 ppb), and 4 meals/month (23–47 ppb).

**Figure 4:**
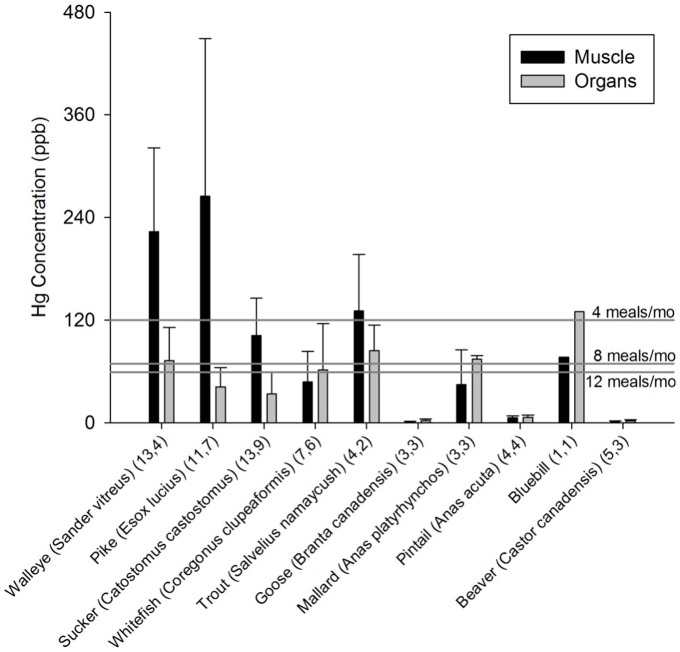
Mercury (Hg) concentration (ppb or ng/g fresh weight) in muscle and organs of various locally-harvested wild animals generally consumed by community members of Kasabonika and Wapekeka First Nations. Data are presented as means ± SD. Sample sizes are indicated for each species (muscle, organ tissues). Monthly fish consumption limits (U.S. Environmental Protection Agency) are indicated for MeHg. The consumption limits are 12 meals/month (59–78 ppb), 8 meals/month (78–120 ppb) and 4 meals/month (120–230 ppb).

The sampled fish muscle tissue (fillets) had low enough ΣPCB concentrations (>12–16 ppb) to be safe to consume at a frequency of 12 meals per month [61]; Figure 3). The walleye organ samples exceeded the U.S. Environmental Protection Agency guideline of 12 meals per month (>12 ppb). The PCBs in locally-harvested rabbit organs (*Oryctolagus cuniculus*) and duck muscle (*Anas platyrhynchos, Anas acuta*, *Aythya affinis*) were higher than piscivorous fish such as lake trout (*Salvelinus namaycush*; Figure 3) suggesting the influence of local contamination source(s) such as landfills or refuse incineration practices which are widely used in these Boreal communities [62]. Mercury concentrations were sufficiently high in many locally-harvested fish muscle/fillet (Figure 4) that participants from these communities (especially WF2 and WF3, who would consume more than 4 meals per month) often exceeded the U.S. Environmental Protection Agency's monthly fish consumption limits (120 ppb at 4 meals) for MeHg, assuming that more than 95% of mercury in fish was MeHg [63].

### 3.3 Plasma phospholipid fatty acid composition in relation to wild food consumption

**Figure 5:**
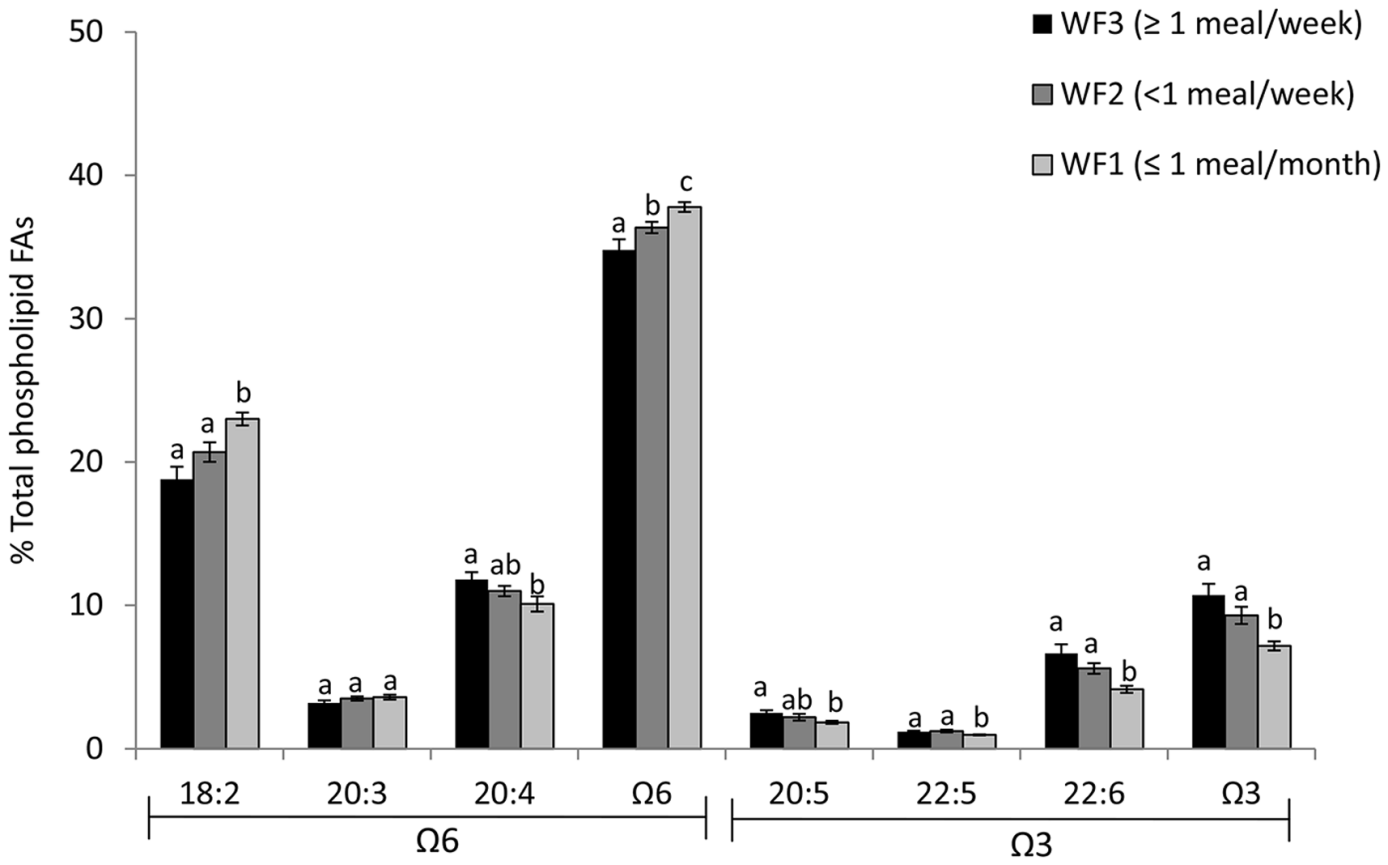
Mean (± SE) contribution of individual fatty acids to total phospholipids in blood (%) for participants from Wapekeka and Kasabonika First Nations consuming three levels of wild food consumption. WF1 (n = 28; ≤1 meal per month), WF2 (n = 22; <1 meal per week), and WF3 (n = 21; ≥1 meal per week) Letters indicate significant differences in groups (p<0.05).

Percent contribution of individual VLC ω-3 fatty acids in plasma phospholipids were related to wild food consumption (Figure 5). The highest and mid-range wild food consumers (WF3 and WF2) had significantly higher mean levels (p<0.01) of VLC ω-3 fatty acids (expressed as a percent of plasma phospholipids) than the WF1 group. The same trend was observed for the docosahexaenoic acid (DHA, 22∶6 ω-3) (p<0.01). The eicosapentaenoic acid (EPA, 20∶5 ω-3) was found to be significantly higher only in the highest wild food consumers (WF3) when compared to the lowest group (WF1; p = 0.01). Conversely, the food group with the lowest rate of wild food consumption (WF1) showed a significantly higher mean level of linoleic acid (LA, 18∶2 ω-6) than both WF2 (p = 0.02) and WF3 (p<0.0001). A similar pattern (p<0.05) was observed for total ω-6 fatty acids where WF1 had the highest mean level of these fatty acids. However in this case, the WF2 group also had a significantly higher mean level of ω-6 fatty acids compared to WF3 (p = 0.04). Fish is the main source of VLC ω-3 in diet [12] whereas linoleic acid, primarily derived from soybean oil, is found in processed store-bought foods [21] [21]. These food items likely explain the different relative concentrations of fatty acids found in plasma phospholipids of the three categories of wild food consumers studied.

### 3.4 Benefits and risks of wild foods

This cross-sectional study provides contaminant and PUFA profiles of communities consisting of a high proportion of frequent wild food consumers. To our knowledge, this is the first study based in the Boreal region of Ontario comparing consumption patterns to both risks (contaminants) and potential health benefits of the diet (PUFA composition of circulating phospholipids). The inclusion of contaminant data based on locally-harvested foods adds strength to the study results.

Overall, we observed significantly higher levels of contaminants in WF3 (consumers of the most wild food) for 10 of 13 contaminants included in analyses. Contaminant levels were significantly different between WF2 and WF1 for only 4 of the 13 contaminants. This result had an interesting contrast to the PUFA results. There was no significant difference between WF2 and WF3 for most PUFAs analyzed. Therefore, although WF3 had an added contaminant burden, no appreciable nutritional benefits from PUFAs were seen compared to the WF2 group. This result should be taken into account when establishing fish consumption advisories for these communities. If consuming fish more than once a month but less than once a week gives the same potential nutritional benefits (through PUFAs), then perhaps an “ideal” frequency of consumption lies between once a month and once a week. Recent evidence suggests that perceived risk of ingesting contaminants through wild food consumption leads many individuals away from these food choices despite their potential nutritional benefits. In the Mohawk community of Kahnawake, a fish consumption decline was reported over a 10 year period based on perception of risk, though sampling local fish found contaminant levels to be relatively low [64]. This research highlighted the need to inform communities about local information on contaminants in food sources in order to encourage informed food choices. Studies in mixed populations have noted misinterpretation and lack of detailed knowledge of risks and/or benefits of fish consumption [65]. The process of risk and benefit communication and emphasizing either the risks or benefits can also have a significant effect on fish consumption frequency [66].

The ω-3 PUFAs, also found in wild foods, have some potential health benefits (such as reduction of carotid artery plaque) that may not be influenced by POPs or Hg [67]. The potentially counteractive effects of POPs against PUFAs in diet on type 2 diabetes and obesity has recently been reported in the mice model [68]. Methylmercury has also been implicated in a higher resting heart rate, even after taking the influence of ω-3 fatty acids into account [69].

Based on the contaminant concentrations measured here, the extent of contamination in locally-harvested wild foods have implications for First Nations peoples who rely on wild foods and value their traditional lifestyles, particularly if individuals were encouraged to consume greater amounts of wild foods for their proposed health benefits. The potential benefits associated with wild food diets must be considered within regional, cultural and environmental contexts. The tremendous diversity of food sources and selection among First Nations/Aboriginal groups [70], [71], must be acknowledged if health benefits are to be assigned to wild foods.

Diet has also been identified as a significant source of exposure to POPs in a global context. In Sweden, fish consumption from the Baltic Sea has been documented to be a source of POPs [72], in Argentina animal and freshwater fish consumption [73] has been identified as an exposure source for organochlorines, and in Japan fish and shellfish consumption [74] has been related to some PCBs and other POPs.

## Conclusions

Advocating the consumption of local wild food resources without acknowledging the regional and environmental diversities of contemporary First Nations populations potentially exposes individuals to health risks. Wild food consumption in excess of 1 meal per week can be accompanied with greater levels of contaminants, without significantly increasing the intake of beneficial ω-3 fatty acids. Overall, this study adds to a growing body of literature reporting diet as a significant source of contaminants that may impact human health. It therefore highlights the need to increase efforts to reduce global contaminant emissions to the environment, particularly for mercury, to curb their transfer to remote northern environments.
